# Cardiovascular Autonomic Neuropathy and Early Atherosclerosis in Adolescent Type 1 Diabetic Patient

**DOI:** 10.3889/oamjms.2015.131

**Published:** 2015-12-13

**Authors:** Soha M. Abd El Dayem, Ahmed A. Battah, Abo El Magd El Bohy

**Affiliations:** 1 *Pediatrics Department, National Research Centre, Cairo, Egypt*; 2 *Critical Care Department, Cairo University, Cairo, Egypt*; 3 *Radiology Department, Cairo University, Cairo, Egypt*

**Keywords:** Cardiovascular autonomic neuropathy, coronary calcification, type 1 diabetic patient

## Abstract

**AIM:**

To evaluate cardiovascular autonomic neuropathy (CAN) in type 1 Diabetics and to detect its relation to coronary artery calcification.

**PATIENTS AND METHODS:**

It is a cross sectional study included 62 diabetics and 30 controls. Clinical, laboratory assessment and 24 Hr holter were done for all patients and controls and coronary artery calcium (CAC) scoring by multisclice CT was done for all patients only. T-test, Mann Whitney U test, and stepwise multiple regression were used for statistical analyses.

**RESULTS:**

CAC score was positive in 8.1 % of patients. Heart rate variability (HRV) was significantly lower in diabetics. All parameters of HRV were insignificantly lower in diabetics with positive CAC score. Patients with microalbuminuria had a significantly lower HRV. HRV had a significant correlation with age of patients, duration of disease, HbA1, and Qtc in diabetics.

**CONCLUSION:**

Percentage of arrhythmia and early atherosclerosis is high in adolescent type 1 diabetic patients. CAN is associated with early atherosclerosis. Cardiac autonomic neuropathy is associated with older age, longer duration, and poor glycemic control and microalbuminuria.

## Introduction

The etiology of cardiac disease in patients with type 1 diabetes (T1D) may involve many factors, including accelerated coronary atherosclerosis, diabetic cardiomyopathy, and cardiac autonomic neuropathy (CAN) [1, 2]. Autonomic neuropathy is a common complication of T1D associated with older age, longer duration of diabetes, worse metabolic control, and the presence of microvascular and cardiovascular disease [3, 4]. Cardiac autonomic neuropathy affects the parasympathetic nervous system, leading to reduced heart rate variability (HRV). In people without diabetes, decreased HRV is associated with subclinical inflammation [5], arterial hypertension [6], and increased incidence of cardiac events [7].

Among the features of diabetic autonomic neuropathy, CAN involvement is of special interest. Diabetic autonomic dysfunction, even when subclinical, is also associated with a high risk of mortality which makes its early identification clinically important. Cardiac dysfunction due to CAN has been demonstrated in diabetic patients without evidence of ischemic heart disease and this can increase the risk of sudden unexpected death [7, 8].

An association between CAN and carotid atherosclerosis, assessed by ultrasound, has been reported in patients with type 2 diabetes [9, 10]. Coronary artery calcium (CAC) is a powerful predictor of clinical coronary artery disease [11]. T1D patients demonstrate greater extent and progression of CAC than subjects without diabetes [7, 8, 12].

We are aiming to evaluate CAN in type 1 Diabetics and to detect its relation to coronary artery calcification.

## Patients and Methods

### Patients

The study included 62 patients with type 1 diabetes mellitus (DM) among those attending to the endocrine clinic, National Research Centre. The control group consisted of 30 ages and sex matched healthy normal volunteers.

Inclusion criteria: Patients with Duration of disease > 5 years, patients age > 14 and < 19 yrs old.

Exclusion criteria: Patients during acute diabetic complications e.g. diabetic ketoacidosis (DKA) or hypoglycemia, patients suffering from cardiac diseases e.g. congenital, rheumatic heart, left ventricular dysfunction, patients receiving drugs for cardiovascular disease.

### Study design and protocol

It is a prospective cross sectional study done after obtaining approval from the ethical committee of the National Research Centre, Cairo, Egypt. Registration number is 11052. Written informed consent was obtained from all patients, their parents and controls after full discussion about the aim of the study. This study is a part from a project done in the National Research Centre in collaboration with Cairo University for evaluation of cardiac, vascular and endothelial function in adolescent type 1 diabetic patients.

All the studied patients were subjected to:

History taking including: Age of patients, sex, age of onset of diabetes, duration of diabetes, type and dose of insulin therapy, family history of diabetes. We asked about presence of any symptoms of cardiac, renal, neurological affection or presence of any type of autonomic dysfunction. We also asked about history of taking drugs other than insulin.

Patients and controls were subjected to general, cardiac, chest and neurological examination.

Simultaneously all patients and controls underwent the following tests:

For cholesterol measurements, venous blood was sampled after 12 hr fast. Serum total cholesterol was determined by a commercial kit (Boehringer-Mannheim, Germany). High-density lipoprotein (HDL) cholesterol was separated from the serum by precipitation of other lipoproteins with a heparin/manganese procedure. Low-density lipoprotein (LDL) cholesterol was calculated using the Friedewald equation. The concentration of triglycerides was measured in a TechnoCon AutoAnalyzer II (TechnoCon Instruments, Tarrytown, New York).

Glycosylated hemoglobin (HbA1) was done every 3 months and the mean value was calculated per year retrospectively from files. It was measured using high-pressure liquid chromatography (Nichols Institute, Van Nuys, California).

Screening for microalbuminuria: It was assessed in fresh morning urine samples by measuring albumin/creatinine ratio by enzyme linked immunosorbent assay (ELISA).

Heart rate variability (HRV) was measured by computerized analysis of long term heart rate samples (1024 beats) using 24 hour holter monitoring space lab boarded in which the software automatically calculates all heart rate indices in a comprehensive and accurate form. Spectral analysis was performed on linearly resample (1 Hz) time series using Welch’s method [13].

Time domain analysis is computed on differing computations of the measurement of the standard deviation of heart period, based on sinus R-R intervals over time [14, 15]. The time domain analysis of heart rate variability can be further divided into two categories. One category is derived from the R-R intervals, using means and standard deviations of the intervals measured in milliseconds. Measures in this category include the SDRR. The SDRR is the standard deviation of all R-R intervals during a 24-hour period. Values for SDRR that are less than 50 milliseconds have been associated with sudden cardiac death [16]. The second category of time domain variables is derived from differences between adjacent R-R intervals and includes indices that are independent of circadian rhythms. The PNN50 is the proportion of the total R-R intervals that have differences of successive R-R intervals greater than 50 milliseconds. SDARR is the standard deviation values of all averaged normal sinus R-R intervals for each 5-minute segment in the 24-hour recordings. The RMSSD is the square root of the mean squared differences of successive R-R intervals. Reflecting alterations in autonomic function that are primarily vagally mediated, the RMSSD, SDRR and PNN50 correlate highly with high-frequency power, reflecting parasympathetic modulation [14]. Other time-domain variables reflect a mixture of parasympathetic, sympathetic, and other physiologic influences [15, 17].

The end of the T wave was defined as the point of maximal change in the slope as the T wave merges with the baseline. QT interval was corrected for heart rate by calculating QTc. QTc was calculated with Bazett’s equation [QTc=QT interval (ms)/√(60/heart rate)] [18].

### Assessment of coronary calcium scoring by multisclice CT

CT examination was performed in one centre using the CT scanner; iCT 256 (Philips Medical Systems; Eindhoven, Netherland). The patient is positioned supine on the CT table. ECG leads are fixed at the four corners of the pericordium. All reconstructions are performed using the retrospective ECG gating. For this technique; an ECG must be recorded simultaneously throughout the duration of the scanning.

First, a localization scan (scanogram) is performed that yields an antero-posterior and lateral views of the chest. It is used to position the imaging volume of the coronary arteries that extends from the level of the carina down to about 1 cm below diaphragm. The center of the field of view is 2 cm to the left of the dorsal spine on the AP scout and at the level of the hilum on the lateral scout.

A non-contrast CT examination of the heart was performed for all patients in order to detect and quantify coronary calcifications through the volume extended from below the carina to the apex of the heart. Acquisition parameters were ECG gated at 75% of the RR interval, 270 ms gantry rotation, 256 × 0.625 mm collimation, 80 mA, and 120 kV. To minimize the total effective patient radiation dose, this stage of the scanning was conducted with a relatively low tube current. The radiation dose of the CT coronary calcium score, according to this technique, is about 1 mSv, in average. A radiologist read all computed tomography using an interactive scoring system similar to that used by Yaghoubi et al., [19].

### Statistical Analysis

Statistical analysis was conducted using Statistical Package for Social Science (SPSS) program version 15.0 (Chicago, Illinois, USA). t -test for independent variables was used and non parametric (Mann Whitney U) test was used when data was not symmetrically distributed. Stepwise multiple regression analysis was also performed to find an association of SDARR, SDRR, DRR, PNN50 and RMSSD with QTc, age of patients, duration of disease, VLDL and HbA1c which had p value <0.05 in simple correlation analysis (Pearson’s or Spearman correlation).

## Results

The study included 62 patients with type 1 diabetes (31 males and 31 females) and 30 healthy volunteer (15 males and 15 females). All diabetic patients were on intensive insulin therapy regimen.

Comparison between demographic, anthropometric and laboratory data of patients and controls were shown in Table 1.

**Table 1:** Comparison between demographic, anthropometric and laboratory data of patients and controls

Variables	Patients		Controls		P-value
	Mean	SD	Mean	SD	
Age (yrs)	16.32	1.52	16.13	2.63	0.70
***Laboratory data:***					
HbA1 (%)	9.55	1.90	5.43	0.65	**0.0001**
Albumin/creatinine ratio (µg/g creatinine)	78.33	100.65	11.28	4.23	**0.0001**
Total cholesterol (mg/dl)	188.81	63.77	100.54	20.41	**0.0001**
Triglyceride (mg/dl)	103.46	78.29	68.89	28.39	**0.03**
HDL-c (mg/dl)	51.77	20.58	52.21	11.12	0.90
LDL-c (mg/dl)	118.66	47.53	62.50	19.88	**0.0001**

HDL-c: High density lipoprotein- cholesterol; LDL-c: Low density lipoprotein – cholesterol; HbA1: Glycosylated haemoglobin.

Average, maximum and minimum heart rate, SDRR, DRR, SDDRR, PNN50 and RMSSD were significantly lower in diabetics (Table 2). No difference in HRV in diabetic patients was found in relation to sex, P > 0.05 (data not present).

**Table 2:** Comparison between parameters of 24 hr holter of diabetic patients and controls included in the study

Variables	Patients Median Mean ± SD (Interquartile range) N = 62	Controls Median Mean ± SD (Interquartile range) N = 30	Test t/Z	P-value
SDARR (ms)	32.0 32.0 ± 12.1 (24.0 – 32.0)	33.0 34.8 ± 10.6 (29.0 – 43.0))	-2.4	0.002#

Average HR (BPM)	90.5 ± 10.2	86.4 ± 8.0	1.85	0.04

Maximum HR (BPM)	163.1 ± 14.8	132.6 ± 18.1	8.2	0.0001

Minimum HR (BPM)	57.3 ± 10.4	67.9 ± 6.8	-4.9	0.0001

Mean RR interval	611.0 ± 79.7	631.6 ± 92.6	-1.06	0.2

Median RR interval	609.1 ± 79.6	629.4 ± 91.9	-1.05	0.3

Standard deviation RR (SDRR) (ms)	33.5 34.4 ± 13.4 (24.0 – 42.8)	41.0 46.6 ± 19.5 (38.0 – 57.0)	-3.0	0.003#

Mean difference RR (DRR)(ms)	22.0 24.6 ± 11.2 (16.0 – 33.0)	32.5 36.1 ± 24.4 (21.3 – 44.5)	-2.4	0.02#

Standard deviation difference RR (SDDRR)(ms)	18.0 37.5 ± 132.7 (13.0- 24.8)	27.0 30.4 ± 20.3 (17.3 – 38.3)	-2.6	0.009#

Percentage of RR > 50 msec (PNN50) (%)	11.0 11.9 ± 8.6 (6.0- 18.0)	17.0 20.3 ± 16.5 7.0- 34.8)	-3.1	0.05#

The square root of the mean squared difference of successive R- R interval (RMSSD)(ms)	27.1 29.5 ± 15.0 (19.0 – 40.0)	44.0 47.3 ± 31.6 (28.0 – 58.0)	3.5	0.002#

Corrected QT interval (QTc)(ms)	381.0 ± 29.2	371.0 ± 43.8	0.47	0.6

ST segment deviation	0.9 1.0 ± 0.4 (0.7 – 1.3)	1.0 1.0 ± 0.4 (0.7 – 1.2)	-0.1	0.9#

t- independent test.

# Mann Whitney –U test.

Five (8.1%) patients had positive coronary calcium score and 28 (41.2%) patients had microalbuminuria. Although all parameters of HRV were lower in diabetics with positive CAC score, it was statistically not significant (Table 3). Table 4 showed the arrhythmia detected by 24 hr holter of patients included in the study. DRR, PNN50 and RMSSD were significantly lower in microalbuminuric patients (Table 5). Triglyceride had as significant positive correlation with minimum and maximum HR (r = 0.4, P = 0.004 and r = 0.3, P = 0.03 respectively). Age of patients (β = -3.2, 95% CI: -5.4 – 1.0, p=0.006) and QTc (β= -0.1, 95% CI: -0.2 - -0.003, p = 0.04) were related to SDARR by stepwise multiple regression analysis. On the other hand, Duration of disease (β = - 1.5, 95% CI: -2.6 - -0.4, p=0.01) and (β = -1.7, 95% CI: -2.9 – - 0.4, p=0.008) and HbA1 (β = -2.2, 95% CI: -4.0 - -0.5, p = 0.01) and (β = -2.5, 95% CI: -4.4 - -0.7, p = 0.009) were related to SDRR and RMSSD by stepwise multiple regression analysis respectively.

**Table 3:** Comparison between parameters of 24hr Holter of diabetic patients in relation to coronary calcium score

Variables	Coronary calcium score			

	Negative Median Mean ± SD (Interquartile range) N = 57	Positive Median Mean ± SD (Interquartile range) N = 5	Test t/Z	P-value	
SDARR (ms)	32.0 32.2 ± 12.7 (22.5 – 41.0)	35.0 31.7 ± 6.7 (24.0- 36.0)	-0.04	0.9#	

Average HR (BPM)	90.3 ± 9.8	85.5 ± 11.1	0.9	0.4	

Maximum HR (BPM)	162.8 ± 14.5	160.0 ± 15.9	0.37	0.7	

Minimum HR (BPM)	57.7 ± 10.5	50.5 ± 8.4	1.34	0.9	

Mean RR interval	616.0 ± 82.5	574.5 ± 36.6	0.09	0.3	

Median RR interval	614.2 ± 82.5	572.8 ± 38.5	1.84	0.3	

Standard deviation RR (SDRR)(ms)	33.5 34.8 ± 14.0 (24.0- 44.0)	33.0 31.8 ± 5.7 (25.8 – 36.5)	-0.3	0.7#	

Mean difference RR (DRR)(ms)	22.0 24.8 ± 11.7 (15.0 – 33.3)	21.8 23.1 ± 6.7 (17.5 – 30.1)	-0.2	0.9#	

Standard deviation difference RR (SDDRR)(ms)	18.0 39.9 ± 140.4 (12.5 – 25.1)	17.5 17.4 ± 4.2 (13.6 – 21.0)	-0.3	0.8#	

Percentage of RR > 50 msec (PNN50)(%)	11.1 12.2 ± 8.8 (6.0 – 18.0)	11.5 11.3 ± 6.1 (5.3 – 17.0)	-0.2	0.9#	

The square root of the mean squared difference of successive R- R interval (RMSSD) (ms)	27.5 30.1 ± 15.3 (18.3 – 41.0)	27.5 28.8 ± 10.1 (19.8 – 39.0)	-0.1	0.9#

Corrected QT interval (QTc) (ms)	381.9 ± 28.6	377.0 ± 29.6	0.33	0.7

ST segment deviation	0.9 0.9 ± 0.4 (0.7 – 1.3)	1.1 1.1 ± 0.2 (0.9 – 1.3)	-1.1	0.3#

t- independent test

# Mann Whitney –U test.

**Table 4:** Arrhythmia detected by 24 hr holter of patients included in the study

Variables	N	%
Ventricular ectopia:		
Number	14	23.3
Form of ventricular ectopia:		
Isolated mono	10	71.4
Isolated poly	4	28.6
Supraventricular ectopia:		
Number	10	16.7
Supraventricular tacchcardia	2	3.3
Sinus tachycardia	1	1.6
Exit sinus block	2	3.3

**Table 5:** Comparison between parameters of 24hr holter of diabetic patients in relation to microalbuminuria

Variables	Microalbuminuria			

	Normalbuminuria Median Mean ± SD (Interquartile range) N = 34	Microalbuminuria Median Mean ± SD (Interquartile range) N = 28	Test t/Z	P-value	
SDARR(ms)	33.0 34.8 ± 10.6 (29.0 – 43.0)	29.0 28.6 ± 13.2 (16.8 – 36.5)	-1.7	0.08#	

Mean RR interval	622.2 ± 81.3	597.2 ± 77.0	1.17	0.2	

Median RR interval	619.4 ± 81.5	596.5 ± 77.1	1.07	0.3	

Standard deviation NN (SDRR) (ms)	37.0 37.4 ± 14.1 (29.0 – 44.0)	31.0 (30.8 ± 11.7 (21.0 – 37.5)	-1.7	0.08#	

Mean difference NN (DRR) (ms)	29.0 28.1 ± 12.0 (19.0 – 34.0)	32.50 20.1 ± 8.4 (13.3 – 23.8)	-2.5	0.01#	

Standard deviation difference NN (SDDRR) (ms)	21.0 22.2 ± 11.5 (14.0 – 25.0)	17.0 56.3 ± 198.8 (11.0 – 21.0)	-1.7	0.09#	

Percentage of RR > 50 msec (PNN50) (%)	14 14.7 ± 9.3 (9.0 -19.0)	7.1 8.4 ± 6.3 (4.0 – 12.6)	-2.7	0.008#	

The square root of the mean squared difference of successive R- R interval (RMSSD) (ms)	31.0 34.5 ± 16.1 (22.0 – 41.0)	22.0 23.2 ± 10.7 (14.0 – 27.9)	-2.9	0.003#

Corrected QT interval (QTc) (ms)	375.2 ± 29.7	388.6 ± 27.2	-1.72	0.09

ST segment deviation	0.9 0.9 ± 0.4 (0.6 – 1.3)	0.9 1.0 ± 0.4 (0.8 – 1.1)	-0.6	0.6#

t- independent test

# Mann Whitney –U test.

DRR was related to VLDL (β = - 0.9, 95% CI: 0.4 – 1.3, p = 0.002) and HbA1 (β = -2.2, 95% CI: -4.3 – 0.1, p = 0.04). On the contrary, duration of disease (β = -1.1, 95% CI: -1.8 - -0.3, p = 0.008) was the only parameter related to PNN50 by stepwise multiple regression analysis (Table 6).

**Table 6:** Stepwise multiple regression analysis of SDARR, SDRR, DRR, PNN50 and RMSSD in relation to demographic and laboratory data

Variables	Β	95% Confidence interval	P-value
*SDARR:*			

Constant	126.5	71.7 – 181.2	0.0001

Age of patients (yr)	- 3.2	-5.4 – 1.0	0.006

QTc	-0.1	- 0.2 - - 0.003	0.04

R^2^ = 0.22, SEM = 11.0			

*SDRR:*			

Constant	69.6	48.1 – 91.1	0.0001

Duration of disease (yr)	- 1.5	-2.6 - - 0.4	0.01

HbA1 %	-2.2	-4.0 - - 0.5	0.01

R^2^ = 0.9, SEM = 11.6			

*DRR:*			

Constant	28.9	11.1 – 46.8	0.004

VLDL (mg/dl)	- 0.9	- 0.4 – 1.3	0.002

HbA1 (%)	-2.2	-4.3 – 0.11	0.04

R^2^ = 0.53, SEM = 5.5			

*PNN50:*			

Constant	22.0	14.3- 29.6	0.0001

Duration of disease (yr)	-1.1	-1.8 - - 0.3	0.008

R^2^ = 0.13, SEM = 8.2			

*RMSSD:*			

Constant	69.5	46.2 – 92.9	0.0001

Duration of disease (yr)	-1.7	-2.9 - - 0.4	0.008

HbA1 (%)	-2.5	-4.4 - - 0.7	0.009

R^2^ = 0.20, SEM = 12.50			

R^2^: Coefficient of determination SEM = standard error of mean. Dependent variables: SDARR, SDRR, DRR, PNN50 and RMSSD. Independent variables: duration of disease, age of patients, QTc, HbA1, VLDL

No significant difference of demographic and anthropometric data, blood pressure, and lipid profile was found in diabetic patients in relation to CAC score.

## Discussion

In the current study, average, maximum and minimum heart rate, SDRR, DRR, SDDRR, PNN50 and RMSSD were significantly lower in diabetics. Reflecting alterations in autonomic function that are primarily vagally mediated, the RMSSD, SDRR and PNN50 correlate highly with high-frequency power, reflecting parasympathetic modulation [14].

Within the pediatric literature, HRV (a measure of cardiovascular autonomic function) was lower in adolescents with T1DM compared to healthy controls [15, 20, 21] and lower in youth with T2DM versus T1DM [22]. Chen et al., [23], reported that, time domain is thought to be a marker of parasympathetic function, and these findings suggest that it can be detected early before any manifestation of symptoms and when conventional tests are still normal.

It also suggests that parasympathetic autonomic dysfunction is the first abnormality to arise in the development of CAN [24]. Time domain analysis of HRV, has several advantages over conventional techniques. In addition to being a more sensitive measure of autonomic dysfunction, it is easy to perform with limited specialist training, does not require expensive and cumbersome equipment, is very quick to carry out, and *4*) is not affected by subject variability [24].

No difference in HRV was found between in males and females in our study. These coincide with the result of Rodrigues et al., [7]. The EURODIAB IDDM Complications Study also found no higher risk of CAN in women [4]. Thus, reduced HRV is not likely to be the basis of the more adverse effect of diabetes on coronary artery disease in women than in men. In the contrary, In the Pittsburgh Epidemiology of Diabetes Complications Study, women with T1D had higher risk of CAN than men [7, 25, 26].

In the present study, 5 (8.1%) patients had positive CAC score and all parameters of HRV were lower in diabetics with positive CAC score, but it was statistically not significant. This may be related to the small number of diabetics and if the number increase it may be significant. In the contrary, Salem et al [27], found that 12 diabetic patients (20%) has positive CAC. This may be related to the selection of patients as they selected patients with duration of disease ≥ 10 yr (longer duration than our patients).

Rodrigues et al., [7], studied young patients with asymptomatic atherosclerosis; they suggest that reduced HRV could be associated with early atherosclerosis rather than being an effect of the ischemia that occurs in established coronary artery disease [7]. They reported that reduced HRV prospectively predicts progression of CAC, a powerful marker of cardiovascular disease risk, in patients both with and without T1D and independently of known cardiovascular disease risk factors. Although the association between CAN and increased cardiovascular mortality in patients with diabetes is known, silent coronary artery disease in diabetes appears to be related to accelerated atherosclerosis than to CAN [2]. However, sympathetic denervation may cause dedifferentiation of vascular smooth muscle cells [28]. These alterations are associated with extracellular matrix production and migration to the intima, changes that have been seen in atherosclerosis [29]. Therefore, the possibility that autonomic dysfunction is implicated in the atherosclerosis process is plausible [7]. Lower HRV was a predictor of CAC progression independent of inflammatory markers. Experimental findings suggest that the nervous autonomic system could significantly modulate inflammatory reaction [7, 30, 31].

In our study, fourteen (23.3%) of diabetics had ventricular ectopia, 10 (16.7%) patients had supraventricular ectopia, 2 (3.3%) patients had supraventricular tachycardia and 2 (3.3%) patients had exit sinus block. The mechanism by which autonomic neuropathy causes increased mortality is unclear. It may be that increased mortality is due to an increased likelihood of cardiac ventricular arrhythmia [32]. Several studies show that the sympathetic nervous system promotes cardiac arrhythmias such as ventricular tachycardia and ventricular fibrillation [33, 34]. The parasympathetic nervous system is believed to play a protective role, decreasing the likelihood of malignant arrhythmia [33, 35].

In the current study, 28 (41.2%) patients had microalbuminuria and the remaining patients were normoalbuminuric. DRR, PNN50 and RMSSD were significantly lower in microalbuminuric patients in the present study. A clinical association between diabetic autonomic neuropathy (DAN) and nephropathy in diabetes has been documented in some studies [7, 36]. Hyperglycemia may be a common factor in the pathogenesis of these complications, and poor glycemic control has been demonstrated to influence the development of progression of both nephropathy and neuropathy. The association between DAN and nephropathy might have a pathogenetic significance rather than being due to the simple coexistence of the 2 diabetic complications [23]. In the contrary, Faulkner et al., [15], reported no statistically significant associations between measures of HRV and albumin / creatinine ratio in adolescent’s diabetics. This may explain that CAN precede the occurrence of nephropathy [4].

Zander et al., [37], investigated the prevalence of CAN in patients with insulin dependant diabetes mellitus at different stages of diabetic nephropathy and found that CAN was more prevalent in patients with diabetic nephropathy, more so as nephropathy progresses. The proposed mechanism by which autonomic neuropathy affecting renal function were modifying 24 h blood pressure profile so that the relatively higher nocturnal blood pressure values cause an increase albumin excretion rate during the night, impairment of renal sympathetic innervation play a role in hemodynamic alterations involved in the early stages of diabetic nephropathy and albuminuria [8].

In the current study, triglyceride had a s significant positive correlation with minimum and maximum HR (r = 0.4, P = 0.004 and r = 0.3, P = 0.03 respectively). This indicates that hyperlipidemia may lead to affection of heart rate which is an early signs of cardiac autonomic neuropathy. The Pittsburgh Epidemiology of Diabetes Complications Study III [25] found on multivariate analysis that increased LDL cholesterol levels and decreased HDL levels were independently associated with the presence of autonomic neuropathy in 168 subjects with insulin-dependent diabetes mellitus. In diabetics, the abnormal HRV is probably secondary to the metabolic derangements of diabetes, decreased capillary perfusion, and hypercoagulability within autonomic nerves and the myocardium, which may be associated with or lead to elevated cholesterol levels [38].

Age of patients (β = - 3.2, 95% CI: -5.4 – 1.0, p = 0.006) and QTc (β = -0.1, 95% CI: -0.2 - -0.003, p = 0.04) were related to SDARR by stepwise multiple regression analysis. On the other hand, Duration of disease (β = - 1.5, 95% CI: -2.6 - -0.4, p=0.01) and (β = -1.7, 95% CI: - 2.9 – - 0.4, p = 0.008) and HbA1 (β = -2.2, 95% CI: -4.0 - -0.5, p = 0.01) and (β = -2.5, 95% CI: -4.4 - -0.7, p = 0.009) were related to SDRR and RMSSD by stepwise multiple regression analysis respectively. DRR was related to VLDL (β = - 0.9, 95% CI: - 0.4 – 1.3, p = 0.002) and HbA1 (β = -2.2, 95% CI: -4.3 – 0.1, p = 0.04). On the contrary, duration of disease (β = -1.1, 95% CI: -1.8 - -0.3, p = 0.008) was the only parameter related to PNN50 by stepwise multiple regression analysis. This coincide with previous studies which demonstrated associations between reduced HRV and older age, extended disease duration (DD), higher HbA1, elevated albuminuria, higher fibrinogen, and increased CAC volume at baseline in T1D subjects [39, 40]. CAN in diabetes is thought to occur at least in part due to the effect of hyperglycemia through the accumulation of reduced sugars in diabetic nerves. The neuropathogenic effect may then be triggered by hyperosmolarity, a change in the reductive–oxidative state, reduced nitric oxide synthesis, or vascular dysfunction [41].

The QT interval represents the time required for completion of both ventricular depolarization and repolarization and has been a parameter of particular interest [42]. This interval is equivalent to the ventricular refractory period as an indicator reflecting myocardial depolarization and repolarization, and is affected by chronotropic changes [43]. Moreover, because the QTc interval duration was related with sympathetic overactivity, QTc prolongation could be an expression of the impaired autonomic activity that characterizes the insulin-resistant subjects with impaired fasting glycaemia and impaired glucose tolerance [43].

No significant difference of demographic and anthropometric data, blood pressure, and lipid profile was found in diabetic patients in relation to CAC score (data not present). It may be related to the small number of positive cases, if the number of patients increases and we select patients with longer duration of disease, we can find more patients with positive CAC score and a significant difference could be found. In the contrary, Salem et al, [27] reported that diabetics with positive CAC had significantly elder age, longer duration and higher mean glycosylated hemoglobin, blood pressure, albumin/creatinine ratio and serum lipids.

*Limitation of the study:* Number of patients with positive CAC score is small. Urine morning sample is used for diagnosis of microalbuminuria as albumin is excreted in urine periodically.

We conclude that Percentage of arrhythmia and positive CAC score was high in our patients. Diabetic patients had reduced HRV. Cardiac autonomic neuropathy is associated with older age, longer duration, and poor glycemic control and microalbuminuria.

We recommend frequent follow up of asymptomatic diabetic patients for early diagnosis of CAN and subclinical cardiovascular disease by CAC score for early diagnosis and management of atherosclerosis.
